# Bridging the Older Adult Digital Divide Through the Digital Habitus

**DOI:** 10.1155/jare/6656355

**Published:** 2026-01-17

**Authors:** Yu Yue, He Langjie, Lai Chi Yuen, Hong Wang Fung

**Affiliations:** ^1^ Department of Social Work Hong Kong Baptist University Hong Kong China hkbu.edu.hk; ^2^ Department of Social Work and Social Administration The University of Hong Kong Hong Kong China hku.hk; ^3^ The School of Nursing The Hong Kong Polytechnic University Hong Kong China polyu.edu.hk; ^4^ Mental Health Research Centre The Hong Kong Polytechnic University Hong Kong China polyu.edu.hk

**Keywords:** digital capital, digital divide, digital habitus, older adults

## Abstract

This study investigates how older adults in Guangzhou navigate a rapidly digitalizing city through the lens of Bourdieu’s habitus and capital. The study draws on semistructured interviews with 25 smartphone‐owning adults aged 60+. Guangzhou’s older adults follow path‐dependent adaptation. Many use smartphones to continue familiar habits, while others adopt new routines. Yet, resistance persists when low education, limited resources, or fear of fraud reduce confidence. Adaptation is partial and uneven, with new practices layered onto old ones and strongly shaped by social position and intergenerational support. We conceptualize digital habitus as embodied dispositions toward technology and digital capital as access plus competencies that convert into economic (time/money savings), social (maintained/expanded networks), and cultural (media engagement) capital. Intergenerational support was a key strategy to bridge exclusion. The study contributes to Bourdieu’s habitus theory by specifying when and how later‐life dispositions adapt in digital fields. The study’s originality also situates digital aging in urban China’s smart‐city governance. Findings inform inclusive design and training that build on existing routines to widen meaningful digital benefits.

## 1. Introduction

The rise of Web 3.0, artificial intelligence, and digitally mediated public services has made digital competence central to learning, social participation, and everyday life [1–3]. Yet the expansion of digital infrastructures has not eliminated inequality. Scholars note that digital resources remain unevenly distributed, allowing the privileged to benefit while others remain excluded [4, 5]. This digital divide is particularly pronounced among older adults. In China, only 52.1% of people aged 60 and above use the internet, which is far below the national average of 79.7%. Many of them still lack the skills and confidence needed to navigate digital environments [6, 7]. Their use of advanced online functions is limited; for example, fewer than 12% use e‐health services despite their relevance for later‐life well‐being [8].

Older adults are vulnerable to digital inclusion. The global aging trend coincides with the persistence of the digital divide, raising questions of equity in later life. In China, the internet penetration rate among people aged 60 and above was 52.1% in 2025, compared to the national average of 79.7%. Nearly half of non‐Internet users belong to this age group, despite representing only one‐third of the total population [6, 9]. Older adults also report significantly lower digital literacy levels than younger cohorts [7]. In terms of usage, their engagement with advanced online functions remains limited: fewer than 12% use e‐health platforms for disease consultation and counseling, even though such services are closely linked to their health and well‐being [8]. Despite national policies designed to narrow the gap, older adults in Mainland China remain disadvantaged in terms of access, skills, and meaningful use of information and communication technologies (ICTs). Guangzhou was selected as the research site because it represents both the opportunities and challenges of digital aging in contemporary China, particularly the pandemic (Zhao, 2024). Guangzhou provides a strategically important context for examining these issues. As one of China’s leading smart‐city pioneers, the city has rapidly digitalized its public services, from transportation to healthcare and social welfare [10, 11]. While such initiatives create new opportunities, they also increase the risk of exclusion for older adults who lack access, skills, or support. Generational differences exacerbate these barriers: many older adults are “digital immigrants” who encounter ICTs late in life, often with low self‐efficacy and uncertainty when engaging with essential services such as online banking and medical appointment systems [12, 13].

Recent scholarship argues that beyond structural inequalities, older adults’ dispositions, attitudes, and comfort toward technology are shaped by lifelong social, cultural, and educational experiences [14]. This points to the relevance of a Bourdieusian framework. Digital habitus refers to the embodied dispositions and routines individuals develop toward digital tools, shaped by past experiences and social position. Digital capital captures the digital access, competencies, and resources that individuals can mobilize for social, cultural, or practical benefits [15]. While these concepts have gained traction in studies of digital inequality (Calderon Gomez, 2021; [16]), they have rarely been applied to the experiences of older adults, and even less so in non‐Western contexts undergoing rapid digitalization. This study fills this gap by examining how digital habitus and digital capital emerge, transform, or stagnate among older adults in Guangzhou. We investigate (1) how older adults develop embodied digital dispositions, (2) how accumulated digital capital translates into real‐life outcomes, and (3) how these processes contribute to either bridging or reinforcing the digital divide. The study situates the analysis within China’s specific sociocultural context, including collectivism, filial piety, and attitudes toward aging. The study extends existing research that has been predominantly in the West. Overall, this article contributes to digital divide scholarship by demonstrating how habitus‐based explanations illuminate the unequal patterns of digital aging in urban China. Three research questions guide this study as follows:

1. How is digital habitus formed, sustained, or resisted among older adults in Guangzhou?
2. How does accumulated digital capital influence their real‐life experiences and outcomes?
3. How do these processes contribute to the persistence or reduction of the digital divide in later life?

## 2. Literature Review

### 2.1. Habitus and Capital

According to Bourdieu [17, 18], habitus refers to the durable systems of dispositions that are informed by an individual’s past experiences, social background, and conditions of upbringing. Habitus is not a set of random choices; it systematically structures how individuals perceive the world, interpret social cues, and act within different fields of life [19]. It is the product of social structures and the mechanism by which those structures are reproduced. People’s social positions influence their attitudes and habits, which guide how they act and make choices, and these often reinforce the social order [18]. These dispositions are not only in the mind but also in the body. They show up in tastes, gestures, routines, and attitudes, which are deeply ingrained and last for a long time [18]. As a result, people’s patterns of thought, feeling, and action tend to display regularities that mirror the material and symbolic conditions of their lives [20]. Therefore, habitus explains why individuals within similar social positions often share comparable orientations and why social inequalities are persistently reproduced across generations. Since Bourdieu’s introduction of habitus, the concept has been criticized a lot for its potential determinism [21]. However, Bourdieu does not claim that habitus is not changing in any way [22]. Although habitus is formed based on past and current social circumstances, habitus can adapt when individuals enter a new field [21]. Such possible changes are not incompatible with the persistence of the habitus, for even when entering a new realm that is different from the original conditions in which people have grown up and lived for a long period of time, the habitus that has previously been formed is not completely discarded or changed. Habitus is constantly reconfigured by the intersection of the individual and the outside world. When people encounter an unfamiliar field, there is a mismatch between the habitus and the field, but some groups can use their reflexive ability to change the habitus and adapt to the new field [21]. Reay et al. [23] show that people can reflect on and gradually adjust their practices [23], although critics argue that Bourdieu’s notion of reflexivity remains underdeveloped [21].

Another concept that often appears with habitus is capital [24]. For Bourdieu, each social actor’s behavior and action are not independent acts being separated from their environmental demands (e.g., cultural codes or norms), social structures (for example, institutional guidelines), or mental conditions, but rather an interacting, entangled, and integrated whole [25]. Therefore, the amount of capital within each field an individual is able to leverage determines their own position in the field [22]. According to Bourdieu [17], three fundamental types of capital constitute the capital theory. The first one is social capital, which concerns the networks, trust, social norms, and connections that the individual is set within and can be mobilized to generate advantages. Second, economic capital is determined by income and wealth, and it exerts influence on other levels of capital that a person possesses. The third and most well‐known form is cultural capital. It refers to cultural resources that people can use in social life. Cultural capital has three forms. In the embodied state, it appears as skills, tastes, manners, and ways of thinking. People gain these through learning and social experience. In the objectified state, it appears as cultural goods such as books, artworks, or digital devices. These objects can be owned and exchanged, but people need knowledge to use them well. In the institutionalized state, it appears as formal qualifications (e.g., diplomas or academic degrees). These give social recognition and value. Bourdieu [17, 18] explained that all types of capital (i.e., economic, social, and cultural) can be converted into one another and accumulated. For example, money can be used to buy education, which becomes cultural capital. Social connections can help a person access cultural goods. In every field, people try to build and exchange capital to gain success, following the “rules of the game” in that field [22].

### 2.2. Digital Habitus and Digital Capital

Although Bourdieu’s concepts of habitus and capital were not originally developed for the digital context, scholars have shown their strong applicability in digital research [25]. Building on this foundation, recent studies introduce digital habitus and digital capital to explain how offline social inequalities and dispositions shape digital engagement and the digital divide [26, 27]. Digital habitus extends Bourdieu’s notion of habitus to the digital sphere. It refers to the embodied dispositions that guide how individuals perceive, interpret, and act within online environments. It captures how people’s long‐held values, preferences, and competencies shape their ways of using and understanding technology. Digital capital refers to the resources that enable effective participation in digital life, encompassing both material access (devices and internet connectivity) and digital skills or literacies [15]. Scholars further identify a third‐level digital divide, which is the differences in the outcomes of digital engagement, such as impacts on health, employment, or social ties [28–30]. Therefore, digital capital encompasses the ability to connect and to translate digital participation into tangible benefits [31]. From a Bourdieusian perspective, habitus underscores the purposive and knowledgeable actions individuals carry out to achieve desired goals [32, 33]. Applying this logic, digital habitus and digital capital allow us to understand patterned practices in the digital realm beyond a simple dichotomy of “traditional versus modern” or “offline versus online” uses [34, 35]. Moreover, the interdisciplinary nature of Bourdieu’s theory aligns with ICT studies, providing both a conceptual bridge and a methodological tool for empirical research [25]. In this connection, older adults’ digital behaviors are structured by their social backgrounds, rationalities, and cultural orientations. Digital resources (e.g., smartphones, online platforms, or information networks) are mobilized to fulfill personal and social goals. Thus, examination of the digital divide through habitus and capital reveals how past experiences shape current technological practices and how these dispositions may persist or adapt under new digital conditions [21, 23].

This study conceptualizes digital habitus as older adults’ embodied dispositions toward technology, reflected in their motivations to use ICTs, preferred communication modes (e.g., phone calls vs. instant messaging), perceived benefits, and confidence in adopting new digital tools. Beyond individual factors, we also consider mezzo‐ and macrolevel influences, which are familial expectations, institutional arrangements (e.g., telecom companies), and cultural norms in regulating or transforming digital engagement (e.g., moral norms around face‐to‐face interaction versus online contact). These ingrained cultural codes become visible through everyday digital routines, shaping how older adults in Guangzhou internalize or resist digital practices. Digital capital is examined through the resources older adults accumulate and deploy (e.g., access to devices and digital literacy) and the ability to convert these assets into meaningful outcomes. These operationalizations connect Bourdieu’s framework with the lived realities of older adults’ digital lives. Finally, habitus tends to reproduce existing dispositions; it can also be restructured when individuals encounter a new field with unfamiliar rules [23, 24]. This transformation is not automatic. It emerges when older adults experience a misalignment between established orientations (e.g., thrift, preference for face‐to‐face communication, and distrust of digital mediation) and the digital field’s logic of speed, connectivity, and abstraction. The friction leads to adaptation or withdrawal, subject to their ability to mobilize economic capital (affording devices and data), cultural capital (digital literacy and confidence), and social capital (support from family and peers).

### 2.3. Chinese Cultural Values and Family Structure and Older Adults’ Digital Habitus, Digital Capital, and the Digital Divide

The digital practices of older Chinese adults are shaped by a sociocultural context markedly different from Western settings. Chinese culture was rooted in Confucian and Taoist traditions, emphasizing moral self‐cultivation, simplicity, and harmonious living [36]. These long‐standing cultural characteristics can produce a digital habitus oriented toward caution, frugality, and low technological intrusion [37]. It led some older adults to avoid complex digital functions in favor of maintaining an uncomplicated lifestyle. Confucian values of frugality, modesty, and li (ritual propriety) cultivate a preference for norms of appropriate phone use, such as avoiding loud conversations or overt displays of self‐expression [38], contrasting with the more expressive, individualistic digital norms documented in Western contexts. Confucian frugality also contributes to a digital habitus that prioritizes cost‐saving and rejects unnecessary digital spending, which influences preferences for free, traditional media and selective engagement with smartphone apps. In addition, culturally embedded expectations around filial piety and hierarchical family relations uniquely mediate intergenerational digital support. Older adults often rely on children as trusted digital gatekeepers, shaping asymmetric patterns of digital learning and reinforcing dependencies absent from Western nuclear‐family structures [39]. Similarly, China’s collectivist orientation encourages conformity to group norms and attentiveness to state‐sanctioned online content [40], fostering a digital habitus aligned with social cohesion rather than individual autonomy. Finally, the strong cultural preference for aging in place [41], combined with declining mobility in late life, reinforces digital practices focused on maintaining close family ties and managing daily needs within the home environment. Despite the fact that there is a growing body of research on older adults’ characteristics of digital utilization with its outcomes, the research closely interrogating the relationship between the older adults’ habitus as being a dynamic and interacting process (also an outcome of it) within the situational/external contexts is nevertheless underresearched. In addition, studies documenting the interacting forces and older adults’ offline (nondigital) lives are in abundance, capturing older adults’ daily preferences and particular lifestyles or daily routines. For example, Zechner [42] examined retired migrants’ transnational habitus, confirming that late lives are marked by reduced mobility and shrinking economic capital, as rising health costs and declining pensions constrained their opportunities. Moreover, Newman et al. [43] demonstrated that older adults’ cultural capital shaped their engagement with modern visual art, while Wangler and Jansky [44] noted that active hobby pursuit and social media (e.g., Facebook) participation with high new media adaptation have characterized older adults’ digital habitus in the Western world. Yet, a unique study examining the Chinese older adults′ digital habitus considering imperatives of “aging in place” and living a “simple lifestyle” while being mobile phone users is still absent.

On the other hand, there is also a strong body of scholarship investigating older adults’ acceptance of digital devices, which, however, did not explicitly adopt the term “habitus.” For instance, Klimova and Poulova [45] and Ma et al. [46] have documented that the perceived usefulness of digital devices, e.g., e‐health or reading online news, together with the perceived easiness of leveraging technological systems and social influences, i.e., maintaining interpersonal relationships, have been crucial explanatory factors motivating older adults to incorporate digital resources into their daily routine and activities. In addition, Kim et al. [47] and Schroeder et al. [48] have summarized the prerequisites that shape older adults’ motivations toward digital practices. They include the knowledge and appropriate prior experiences of digital utilization and the need awareness (for instance, using e‐health); the appropriate demographic mix, high income in particular; the self‐efficacy and self‐determination in accessing technologies; and the physical capacity (e.g., without limits of disabilities) to use them. Meanwhile, social influences, for example, peer support, are all‐powerful predeterminants for older adults’ digital utilization development of habituated digital usage. Furthermore, Foster et al. [49] stated that the most common motif for the older adults’ digital utilization is associated with their healthcare and medical needs, yet some also preferred browsing websites and an e‐calendar for setting reminders. These studies have generated valuable insights for understanding older adults’ preferences and preferred patterns of technological use and the fields in which they are expected to be utilized. Yet, such research has left the ways in which cultural values and social norms have restricted or encouraged the older adults’ utilization of mobile devices much underinvestigated. Thus, in line with the sociocultural tradition of the Bourdieusian research, the current study is motivated to contribute to the digital habitus scholarship through shedding light on the older adults’ cultural values, the situational drive, preference, and mode of digital utilization, together with their translation into differential capitals, which can represent disparity of outcomes in terms of older adults’ digital utilization. The present study is an attempt to expand the digital habitus scholarship, primarily investigating the younger users, such as students or “digital natives,” to the “digital immigrants” of older adults, whose motivation and resources (capitals) on hand and cultural tenets/norms in (re)structuring their differential disposition, inclination, or tendency to foster digital habitus (e.g., [25, 27, 50, 51]) still deserve more nuanced studies before the current issue of older adult digital divide can be resolved. Worse is that, without a deepened understanding of such complex interplay in older adults’ formulation and sustenance of digital habitus, the persistence of older adults’ social exclusion, which is a broader structural inequality than a reductivist view of lacking a physical device [52], will continue to hamper older adults with fewer educational, economic, or health resources, in addition to their hindered social inclusion.

## 3. Methodology

Our research is a qualitative design as a response to the scholars’ call for the urgency of qualitative designs for researching the issue of the digital divide (e.g., [16, 53]) to understand from the voices of users of technology. We have conducted semistructured interviews with 25 older adults aged 60 or above who possess at least a smartphone (computer or tablet). Participants were selected using purposive sampling to capture diversity among older adults in Guangzhou. Given the exploratory nature of this study, rigid sampling quotas (e.g., proportional representation across education or income levels) were not imposed. Instead, broad inclusion criteria were applied to ensure participants occupied social positions relevant to the development of digital habitus and digital capital. Eligible participants were Chinese adults aged 60 or above, possessed a functioning smartphone, and had at least primary‐level literacy, which ensured a basic capacity to engage with digital technologies. Participants were recruited primarily through community centers in Guangzhou, where social workers who regularly serve older residents informed potential participants about the study. In addition, a small number of participants were reached through word‐of‐mouth referrals to include individuals of varying socioeconomic backgrounds. Those who expressed interest participated voluntarily and gave informed consent before the interviews. Data collection took place between 23 June and 12 November 2025. All interviews were conducted face‐to‐face, audio‐recorded with permission, and subsequently transcribed verbatim for coding and thematic analysis. We conduct our data analysis through thematic analysis, which is a method of “identifying, analyzing, and reporting patterns (themes) within data” ([54], p. 80). Throughout the process, each interview was coded using the MAXQDA software. As shown in Table 1, the sample contained an even gender distribution (14 men and 11 women) and an age range from 60 to 93. Most participants were between 60 and 74 years old. Respondents were mainly secondary school graduates (*n* = 11), followed by those with primary education (*n* = 7) and undergraduate degrees (*n* = 7). Income levels also varied: 17 interviewees earned below RMB6000 per month, placing them in the lower‐income bracket relative to Guangzhou’s average older adult income and expenditure levels, while only eight participants reported incomes above RMB6000. Taken together, the diversity across age, education, and income enables generalizing the digital habitus of older adults in China. This study obtained ethical approval from the research ethics committee of a university in Mainland China. All participants were fully informed about the purpose, scope, and procedures of the study before data collection. Written informed consent was obtained. All identifying details have been removed from transcripts and analysis. Audio recordings and transcripts were stored securely on password‐protected devices accessible only to the research team.

**Table 1:** An overview of the demographics of the interviewees.

	Gender	Age	Education level	Income (monthly)	Coliving with family member(s) (yes/no) and no. of other family members
Interviewee 1	Male	80	Primary school	Less than RMB2999	Yes, 1
Interviewee 2	Female	69	Primary school	RMB3000‐5999	Yes, 1
Interviewee 3	Male	74	Primary school	RMB3000‐5999	No
Interviewee 4	Male	93	Primary school	RMB3000‐5999	No
Interviewee 5	Male	92	Secondary school	Less than RMB2999	No
Interviewee 6	Male	75	Secondary school	Less than RMB2999	Yes, 1
Interviewee 7	Female	76	Primary school	RMB3000‐5999	No
Interviewee 8	Male	79	Secondary school	Above RMB9000	Yes, 1
Interviewee 9	Female	63	Secondary school	RMB3000‐5999	Yes, 2
Interviewee 10	Female	79	Primary school	RMB3000‐5999	Yes, 1
Interviewee 11	Female	60	Undergraduate	RMB6000‐8999	Yes, 2
Interviewee 12	Female	61	Undergraduate	Above RMB9000	Yes, 1
Interviewee 13	Female	61		RMB3000‐5999	Yes, 1
Interviewee 14	Female	68	Secondary school	RMB3000‐5999	Yes, 1
Interviewee 15	Female	64	Secondary school	Less than RMB2999	Yes, 4
Interviewee 16	Female	63	Secondary school	RMB3000‐5999	Yes, 1
Interviewee 17	Male	63	Undergraduate	Above RMB9000	Yes, 1
Interviewee 18	Male	67	Undergraduate	RMB6000‐8999	Yes, 1
Interviewee 19	Male	73	Secondary school	Less than RMB2999	Yes, 2
Interviewee 20	Male	70	Secondary school	Less than RMB2999	Yes, 1
Interviewee 21	Male	70	Undergraduate	Above RMB9000	Yes, 1
Interviewee 22	Male	69	Secondary school	Less than RMB2999	Yes, 2
Interviewee 23	Female	68	Secondary school	RMB6000‐8999	No
Interviewee 24	Male	69	Primary school	RMB3000‐5999	Yes, 1
Interviewee 25	Male	81	Undergraduate	Above RMB9000	Yes, 1

## 4. Findings

### 4.1. The Older Adults’ Digital Habitus Illustrated by Smartphone Utilization

#### 4.1.1. Sustaining Old Habitus by Using Smartphones to Perform Conventional Tasks

Several participants (*n* = 5; Interviewees 2, 7, 9, 10, and 19) integrated smartphones into daily activities without fundamentally altering or modifying their accustomed lifestyle, in particular their preference of retaining contacts with family members, relatives, and close friends. Several respondents emphasized their primary phone use for sustaining the interpersonal ties they had long established, while online contacts kept their relatives or friends connected.

“Yes, I have used the mobile phone for calls, WeChat, and contacting friends…(asking in WeChat group) when are you free? Are you free? Come out when you’re free!… If you stop usually messaging people on WeChat, it means we’ve lost touch. If that’s like that, why don’t you meet up sometime when you’re free, go out for a walk, or grab some tea…” (Interviewee 2)

“At that old age, there’s no need to do anything online…it’s much more convenient now. It’s like I could just call your grandson and he’d come to see me…because I don’t live with my son.” (Interviewee 19)

In addition, these seniors primarily adopted smartphone usage for assisting the reproduction or better performance of the conventional cultural practices of viewing TV, attending NGO activities, and listening to traditional music. In other words, it is the change occurring in these fields (e.g., TV news shifting online) that compelled these older adults to adopt digital resources to suit their offline habituated practices. For instance, while talking about entertainment, Interviewee 2 stuck to the traditional Mahjong game: “I have one or two phones with two games on them, like Mahjong or something like that.” Interviewee 7 also preferred face‐to‐face interaction with friends in daily life: “I took a bus to (XXX) to eat with him (the friend). Oh, we ate together again… I thought there was no soup here. Oh, we need soup at night, so we took the soup from the food court and enjoyed it together” (Interviewee 7). Meanwhile, their accustomed routine of daily practices, e.g., face‐to‐face gathering with friends/offspring and grabbing a tea, has remained unchanged. Indeed, these participants are highly satisfied with the existing ways of life and conditions of mobile device use, while such stabilized patterns in late life are unwanted by them. Thus, digital practices with smartphones are taken up only to the extent that they align with long‐standing dispositions formed over a lifetime, being informed by a traditional Chinese culture, as long as they are retained with minimal engagement with unfamiliar technologies. The narrative of “there’s no need to” (by Interviewee 19) also indicated his inclination to live a simpler but not a frugal lifestyle. That is, digital devices are incorporated into everyday life but without reorganizing or reordering their digital practice. The digital capital acquired here (very basic functional use of phones) serves mainly to sustain long‐existing forms of social connection rather than generating new advantages. Therefore, the result is continuity: digital tools are valued as convenient extensions of old traditional practices, but not as gateways to new fields of action. In this sense, it endorses that habitus is durable, in that the digital tools are assimilated into routines without altering underlying orientations.

#### 4.1.2. Minimal Establishment of New Digital Habitus

In contrast to their initial ways of life, 14 of the 22 interviewees reported having established new habits relating to more advanced cellphone usage, which represents discontinuation from their previous habituated patterns of life. The newly built daily routine includes checking phone messages when they wake up, friends’ texts for a gathering, e‐payment, watching short videos, reading news or listening to news and audiobooks, booking medical online appointments, online shopping, and food delivery. Such accounts reflect the gradual formation of a digital habitus, where smartphones and tablets are no longer peripheral but woven into everyday life. For example, certain interviewees recognized a need to learn ICT skills.

“Checking the phone when waking up, to check if my friends have found me … … after dinner watch some videos on the Youku.” (Interviewee 6).

“(Learning to use) Navigation solves the problem for us, making it convenient to find a location.” (Interviewee 13)

These respondents have been relatively engaged in a broader engagement in different fields, e.g., volunteering at district organizations (Interviewee 8) and traveling within China (Interviewees 6, 8, 10, 11, 18, 20, and 21), that imposed situational demands requiring them to leverage digital resources (e.g., checking bus routes and buying tickets). Also, their relatively rich interpersonal network and better educational attainment indicated their immediate resource to cope with problems associated with phone use. For instance, more than half of these older adults sought assistance from children or offspring while encountering problems with phone utilization (Interviewees 1, 8, 11, 12, 14, 16, 18, 20, and 21). Furthermore, these respondents are primarily motivated by a need to keep in contact with the family, especially to keep in touch with offspring or remote relatives to leverage digital resources (e.g., Interviewees 6, 10, 11, 12, 13, 15, and 16), which demonstrated their adherence to the traditional emphasis of big family culture. Although many participants did not use the full range of functions of digital devices, their incorporation of new digital practices into routines shows how embodied dispositions are being reshaped in later life. These older adults are not merely reformulating old schemas (i.e., making phone calls) but transforming their new habitus through interaction between the offline and digital fields. They begin to accumulate digital capital by learning some new skills and recognizing the need for continual learning in digital technology through repetitive practice, and the digital skills they acquire can be converted into social benefits (maintaining contact), cultural enrichment (accessing media), and practical advantages (navigating urban space). These findings revealed that habitus are both durable and adaptable; new technologies can open space for transformation even in later life.

Despite the spread of smartphones into daily life, it is notable that most of these new uses were concentrated in the sphere of entertainment and consumption of digital content rather than more complex digital production or interactive practices. Their worries about online fraud have still been strong to forestall their full integration of the digital world into real life. Interviewee 18 commented: “*Convenience has its pros and cons. For example, for customers, while it’s convenient, the downside is that you might buy counterfeit goods.*” Also, several participants still retained a strong traditional attitude to reject phone users lacking the courtesy in public space, which is tied to traditional culture valuing thriftiness and “li (or courtesy).” As Interviewee 9 put it: *“People seem so busy these days……. It wasn’t like this in the past… even when walking, they’d be glued to their phones…… But when you’re walking, you don’t know what you’re looking at…*” These narratives highlight that certain acculturated habitus remains durable, especially when they are shaped by a lifetime of routines centered on frugality and respecting others. In addition, their perceived identity as “I am old” becomes tied to low digital engagement. This shows how perceived self‐efficacy can lock older adults into marginal positions within the digital field. Thus, these acculturated cultural beliefs and self‐images have kept older adults on the margins of the digital world.

#### 4.1.3. Resistance of Engaging With New Digital Technology

Three participants (Interviewees 3, 4, and 5) expressed resistance to adopting new digital technologies (mostly the low‐income and low‐educated participants), emphasizing that smartphones conflicted with their established ways of acting and social conduct. Yet, such social conduct is, once again, highly consistent with the Confucianist and Taoist notions of leading a simple, down‐to‐earth life. Another common theme was lack of confidence: older adults described feeling that their learning ability was weaker than that of younger people, which discouraged their experimentation.

“Nope…We don’t (use WeChat). There’re few contacts between us (respondent and his friends). It’s just that a few of us brothers (close friends) keep going out, sometimes maybe we go for tea again*…*Nope, I don’t have the courage to learn that (e‐payment) certainly means I don’t have the courage to use it.” (Interviewee 3)

The perceived self‐efficacy acts as a barrier to acquiring digital skills, because the expectation of difficulty discourages experimentation. These older adults perceived themselves as having limited competence that does not align with the demands of the digital field. It revealed a dominant perception that aging itself limits learning capacity, a disposition shaped by broader societal narratives of cognitive decline. Older adults did not see digital engagement as a pathway for new forms of life but framed age as a natural justification for reduced participation.

In addition, fear of risk and scams also reinforces their adoption of resistance. For example,

“Someone said he will give me a phone*…* how could I possibly believe in things like that? So I just deleted them*…* now I just live a very simple life, living with my family.” (Interviewee 3)

“The tele‐sales didn’t say anything, he said like it’s not an online plan. So I’d that determination*…* because I don’t know how to use the internet, I don’t even use the internet*…*but online plan is ¥70 per month, so I just use the traditional one.” (Interviewee 4)

These interviews reveal that older adults are highly cautious online. Many of them recognize scams that exploit friendship online. It violated their traditional belief in genuine and ethical relationships. Another key feature is social isolation. Most participants live alone and lack support when facing digital difficulties. Consistent with the idea of “aging in place,” physical decline also limits their participation in activities such as travel, reducing opportunities that require online engagement, such as booking hotels. In addition, a strong attachment to traditional media has been observed among these older adults who prefer more easily accessible programs, without the need for knowledgeable or skillful manipulation of digital artifacts. As Interviewee 4 put it: *“I don’t have the intention to learn these things (using smartphone), I am old …… I always watch the news through the television, because it is free of charge.”* Furthermore, the focus on television and radio being “free of charge” revealed how older adults’ limited economic resources and lifelong habits of thrift shape their media choices. Unlike the younger generation, who used smartphones for entertainment and consumption, the older adults preferred simplicity in life, which is tied to Confucianist and Taoist dogmas. In the digital field, such resistance also represents a digital habitus of purposive withdrawal, which makes withdrawal bring a sense of security and safety rather than trying new practices. Such a preference makes excessive smartphone usage appear as unnecessary or burdensome.

Altogether, the rejection of technology is also a protective strategy interacting with the limited resources, mixing with their culturalized identity and experiences. In addition, the traditional values of frugality also encouraged older adults to consider affordability in a data plan. In turn, their deficit of digital capital owing to outright rejection of modern technology featuring online scammers, combined with their low socioeconomic status, aligns them with frugality, which has undermined their accumulation of digital knowledge. Also, their relatively deeper social isolation (e.g., Interviewees 3, 4, and 5 are lone older adults at home) has further eroded their motivation to use it while heightening their sense of vulnerability in the digital field. As a result, avoidance of digital practices becomes a protective strategy, although it keeps them from acquiring digital skills.

### 4.2. Implications of Digital Capital on Real‐Life Benefits

In addition to patterns of habitus, our findings also show how older adults’ engagement with smartphones leads to the uneven accumulation of digital capital. Some of the interviewees mentioned specific benefits they enjoyed through the utilization of digital devices or access to the online world. Nevertheless, in terms of the proportion of older adult respondents who succeeded in converting such digital capital into economic capital, it was merely those who are better educated (secondary school level and above) and those with higher levels of income (monthly RMB 6000 and above), which indicated that for those with lower income and less education, the motivation or drive to utilize online banking and financial management apps was reduced by the literacy level or management needs. That is, the possession of a higher economic status is a prerequisite for obtaining a higher level of digital literacy, which implicitly points to the importance of paying attention to older adults’ diversified socioeconomic statuses before engaging them in the e‐finance and online banking realms. Though being the relatively few, the benefits of managing finance through the utilization of phone apps with smartphones’ utilized functionality for e‐payment and e‐banking have abandoned the traditional habitus of lining up and drawing money from the reception staff (they have higher monthly income and are well‐educated, *n* = 6; i.e., Interviewees 8, 11, 12, 13, 23, and 25). They, in turn, were praised for the cost‐efficiency and capital accumulation features that, once again, reinforce their class background even further (economic capital). For example, several respondents have reported accounting for the benefits of accessing information through viewing financial news, reducing their economic pressure postretirement, as well as the great convenience associated with online e‐banking service to reduce the hours spent lining up in the bank simply to take money from the account.

“The financial news is good, but sometimes it loads and loads, maybe the network is problematic…this finance, now can be seen,” “I bought it when it was ¥60, and it resumed dividend payment since last year, I treat it as one of my income sources!” (Interviewee 8)

“Well, going to the bank is such a hassle*…* so now I transfer money to my son online. Walking in and out of the bank takes more than an hour, and even if you do go, it’s done at an ATM. That was a long time ago; now transfers are much faster.” (Interviewee 23)

Such porousness between real‐life fields and the online world also ensures the detrimental outcomes of online scams/crimes, which affect the older adults’ stance concerning digital usage. For example, one shared his worry concerning privacy leakage and digital scams:

“The TV news discussed some online scams. The frauds claimed that they help people to apply for credit cards, claiming they can help you invest and other things*…*” (Interviewee 24)

The comment reflects older users’ sensitivity for self‐protection in a commercialized world, where interests play an important role in ruining interpersonal relationships and financial well‐being.

To add, the use of WeChat also facilitates the sharing of photos and information among older people, improving their interpersonal bonds. WeChat is particularly mentioned as very useful for forging their connection with their peers, which plays a crucial role in their participation in their interest groups and friends’ gatherings. In these cases, digital practices expanded access to cultural goods and reinforced ongoing social roles, demonstrating the convertibility of digital technology into cultural capital. The participants, who are in low‐income, less educated groups, did not expand their social circle using social media; instead, they relied on conventional face‐to‐face meetings to maintain contact with friends and relatives.

### 4.3. Bridging the Older Adult Digital Divide

A number of respondents have stated that they have greater motivations to utilize their phones after tasting the benefits offered by digital access, as experienced by the Interviewees 1 and 12.

“Now the mobile phone is the most important thing, it’s impossible to live without it.” (Interviewee 1)

“Nowadays, you cannot imagine in daily life or travel can do without a smartphone. Look at people going out, whether they’re heading out or staying home, a single phone pretty much handles everything.” (Interviewee 12)

When older adults see the benefits of digital tools, their digital capital grows. They gain skills, confidence, and a more positive attitude toward technology. In addition, the situational demands (e.g., traveling) have further motivated older adults to resort to digital resources for coping, leading to a perceived benefit and satisfaction toward the phone. Such satisfaction automatically fuels their subsequent adoption of digital devices for e‐payment, medical appointments, reserving a ticket, and WeChat conferencing with kids. These incessant uses and positive appraisals become a lively cycle, where the more benefits they experience, the more motivated they become to use smartphones. Over time, this stabilizes their digital habitus, or the way they integrate digital tools in daily life.

Children emerged as the most significant mediators of digital engagement. They provided technical assistance as well as facilitation for older adults to experiment without fear of breaking devices or making mistakes. As one participant explained: *“I used to be afraid to try, I’d worry that pressing the wrong button would break something. I’d just watch them try this and that. Later, I realized how much that affects me”* (Interviewee 11). The intergenerational transfer of knowledge effectively converts social capital into digital capital, bridging gaps in skills and confidence. It shows the critical role of family support in overcoming the digital divide among participants.

## 5. Discussion

This study contributes to digital divide scholarship by examining how digital habitus develops among older adults in urban China and how cultural dispositions shape both the adoption and resistance of digital practices. While prior studies (especially Western ones) emphasize motivations (e.g., maintaining social ties and supporting health needs), this study shows that these factors also apply in Guangzhou. However, the Chinese case reveals additional dynamics of family‐based support, cultural norms, and social inequalities that all interact to enable or constrain digital participation. In the following, we revisit these findings through the lens of capital, habitus, and cultural mediation while also addressing the uneven nature of intergenerational support, as raised by the reviewer.

### 5.1. The Importance of Digital Capital and Its Implications

Another major finding is that digital capital was meaningful not on its own, but in its ability to convert into other forms of capital. Smartphones supported everyday needs tied to health, finance, and social connections. For example, participants used e‐banking to save time and earn extra income (economic capital), social instant message applications to sustain networks (social capital), and online entertainment consumption to stay mentally active (cultural capital). This confirms Bourdieu’s argument that capitals are convertible [17] and supports Calderón Gómez’s [31] claim that digital and offline capital reinforce each other. Another theme that is consistent throughout our findings is that older adults rarely report advanced phone utilization, such as online gaming, attending online health seminars, or even video conferencing, which has much to do with their habituated daily routine and internal understandings of their social positions as older adult people who are not in progression but in decline. Given their limited digital capital, the crux lies in how they can translate most of it into real‐life benefits tied to benefiting other capital outcomes, given the different field demands. Therefore, the utilization of smartphones must be tailored to better cope with their daily challenges, rather than adding hurdles to it (as clearly indicated by the negative appraisal of meeting new friends, which added troubles to their lives). Easy‐to‐use apps, such as Youku and WeChat, have proved to be rather accommodating to their accumulation of knowledge and information (cultural capital) and family networking (social capital). This indicates that the accommodation to the older adults’ digital habitus, being situated into their everyday fields and environmental demands, will be a relatively effortless way to bridge the older adults’ digital divide since digital is only playing a secondary role in achieving the older adults’ life goals. Last but not least, older adults’ digital habitus is not uniform but fragmented. Some participants may have reproduced long‐standing practices, established new digital practices, or resisted change, which shows that sustaining and adapting dispositions coexist, producing diverse trajectories of digital aging. The bridging role of intergenerational social capital is necessary for older adults. Children provide technical assistance and encourage experimentation, enabling older adults to overcome fear and build confidence [55]. Focusing on Guangzhou, this study shows how digital inclusion in urban China reflects both opportunities and inequalities. Smartphones provide access to health, finance, and cultural participation, but benefits are unevenly distributed [56]. The persistence of sustaining and resistant habitus suggests that digitalization does not erase inequalities but reframes them in new domains.

### 5.2. The Important Role of Culture in Forestalling Changes

The findings show that cultural dispositions, such as thriftiness, filial piety, and modesty, strongly shape how older adults translate long‐standing habits into digital practices. Thrift often discouraged experimentation with new or data‐consuming applications, creating tension between traditional values and emerging digital norms. Filial piety sometimes enabled digital adaptation by providing intergenerational guidance, but this support was far from universal. The support from grandchildren tends to be informal and highly contingent on family structure, proximity, and generational dynamics [57]. Grandchildren are rarely primary caregivers, and their assistance is often limited by school commitments, geographic distance, and their own lack of digital or emotional maturity [57]. Even when grandchildren are willing to help, their support can be task‐specific (e.g., helping set up a phone or fix a single problem) rather than sustained digital guidance. Moreover, older adults may feel reluctant to “burden” younger grandchildren, especially when cultural norms emphasize protecting the young from responsibility [58]. As a result, grandchild‐provided support is situational rather than stable. Thus, digital adaptation is relational and uneven, shaped by how cultural meanings are reinforced or reinterpreted under new technological conditions [32, 33].

Digital capital did not convert uniformly into other forms of capital. Only a minority of well‐educated, middle‐class participants were able to mobilize digital literacy for activities such as financial management or entrepreneurial tasks. For the majority with fewer economic and educational resources, digital engagement remained largely instrumental, focusing on communication, simple online searches, or basic service access, but they may not generate significant cultural or economic returns. This demonstrates that the conversion of digital capital into other capital forms is structurally conditioned. It is echoed in Bourdieu’s framework (1986): preexisting economic and cultural capital acted as prerequisites for acquiring and leveraging digital skills, leading to a self‐reinforcing cycle where digital competence amplifies prior advantage rather than redressing inequality. In this sense, digital inclusion alone seldom transforms social hierarchies; without institutional or socioeconomic support, it risks reproducing them.

Not all participants experienced a shift in habitus, but some adapted their routines when cultural values aligned with digital practices. For example, using WeChat to maintain family closeness. In these cases, intergenerational contact supported confidence and gradual learning. By contrast, socially isolated or economically constrained participants often adhered to values of simplicity and frugality, viewing smartphones as unnecessary or incompatible with their vision of old age. Limited exposure and weak support prevented the formation of stable digital routines. Not all participants experienced a shift in habitus, but some adapted their routines when cultural values aligned with digital practices. For example, using WeChat to maintain family closeness. These moments echo Yang’s [21] argument that individuals may exercise agential reflexivity when cultural meanings and new practices converge. In contrast, socially isolated, less educated, or economically constrained participants often adhered to traditional values of simplicity and frugality, viewing smartphones as unnecessary or incompatible with their vision of old age. Limited exposure and weak support prevented the formation of stable digital routines, reinforcing low digital engagement and reduced self‐efficacy [13].

These divergent trajectories illustrate that Chinese cultural values can both facilitate digital engagement and justify digital withdrawal. The ability to transform cultural dispositions into digital practices depends heavily on older adults’ social position, including education, income, and access to supportive networks, highlighting how culture, capital, and perceived position within social fields interact to shape late‐life digital transformation [21].

### 5.3. Practical Implications of Habitus Formation and Continuity in Bridging the Divide

The continuity of digital habitus can help bridge the digital divide among older adults. Many older people use smartphones mainly to stay connected with family and friends, which continues long‐standing cultural values of social closeness in Chinese society. This sense of continuity encourages older adults to use smartphones more frequently, because they recognize the practical benefits to their daily lives. Frequent use gradually improves their ability to produce and share information effectively in digital spaces [59, 60] and boosts their confidence to navigate online environments [61].

As mentioned, cultural forces shape older adults’ offline habits, routines, and the development of their digital habitus. The rigid attachment to traditional culture can hinder technology adoption [62], and these same cultural values can be leveraged to support digital adaptation. Policymakers, ICT educators, and social workers should strengthen familial support systems, provide community‐based ICT help desks, and create opportunities (e.g., subsidized social activities, digital tourism, or online mapping) to increase digital exposure among older adults.

Traditional values of filial piety can motivate children to help their parents overcome social isolation and use health‐related apps, which fosters digital familiarity. Importantly, digital habitus should be sustained by offline cultural resources. A gradual approach that starts from resistance, moving to assisted ICT use, and develops stable digital routines works better than uniform training programs. Tailor‐made interventions are essential to align cultural values with digital participation and promote inclusive aging.

## 6. Conclusion

The study makes three main contributions. First, it identifies the digital habitus of older adults in Guangzhou, showing how existing cultural dispositions continue to shape their engagement with digital technologies. Second, it reveals that the transformation of digital capital into other forms of capital is conditional and unequal, which benefits those who already possess richer economic, educational, or social resources. Third, it enriches Bourdieu’s framework by showing how cultural forces (e.g., filial piety and thrift) mediate the ways in which habitus changes in later life, demonstrating that some dispositions persist while others adapt under digitalization. However, this study has several limitations. The small and nonprobability (purposive) sample limits the generalizability of the findings. The underrepresentation of higher‐income participants and the demographic heterogeneity of the respondents revealed that caution is needed in future studies when applying these results to other subsets of older adults. Second, the qualitative approach captures digital behaviors at a single point in time and cannot show how older adults’ lifestyles and technology use evolve; the future study should be a longitudinal study to examine how digital habits, cultural dispositions, and capital conversion processes change over time.

## Ethics Statement

This study was conducted in accordance with the Declaration of Helsinki and approved by the Institutional Review Board of a University in Hong Kong, China.

## Conflicts of Interest

The authors declare no conflicts of interest.

## Funding

This work received no funding.

## Data Availability

The data are unavailable due to privacy or ethical restrictions.
