# Correction to “Bridging the Older Adult Digital Divide Through the Digital Habitus”

**DOI:** 10.1155/jare/9753271

**Published:** 2026-10-21

Y. Yu, L. He, C. Y. Lai, et al., “Bridging the Older Adult Digital Divide Through the Digital Habitus,” *Journal of Aging Research* 2026 (2026), 6656355, https://doi.org/10.1155/jare/6656355.

In the abovementioned article, the article type was incorrectly stated as “Review Article.” The correct article type is “Research Article.”

This has been corrected in the published version online.

We apologize for this error.

